# Effect of immunosuppression in miRNAs from extracellular vesicles of colorectal cancer and their influence on the pre-metastatic niche

**DOI:** 10.1038/s41598-019-47581-y

**Published:** 2019-08-01

**Authors:** Valeria Tubita, Joan Segui-Barber, Juan José Lozano, Elisenda Banon-Maneus, Jordi Rovira, David Cucchiari, Daniel Moya-Rull, Federico Oppenheimer, Hernando Del Portillo, Josep M. Campistol, Fritz Diekmann, Maria José Ramirez-Bajo, Ignacio Revuelta

**Affiliations:** 1 Laboratori Experimental de Nefrologia i Trasplantament (LENIT), IDIBAPS, Barcelona, Spain; 2 Instituto de Salud Global de Barcelona (ISGlobal), Hospital Clínic, Universitat de Barcelona, Barcelona, Spain; 3 Bioinformatics Platform, CIBEREHD, Barcelona, Spain; 4 Laboratori Experimental de Nefrologia i Trasplantament (LENIT), FCRB, Barcelona, Spain; 5 Spanish Kidney Research Network, ISCIII-RETIC REDinREN RD016/0 009, Madrid, Spain; 6 Institut d’Investigació Germans Trias i Pujol (IGTP), Badalona, Spain; 7 Institució Catalana de Recerca i Estudis Avançats (ICREA), Barcelona, Spain; 8 Department of Nephrology and Renal Transplantation, ICNU, Hospital Clínic, Barcelona, Spain

**Keywords:** Mechanisms of disease, miRNAs

## Abstract

Colorectal cancer (CRC) occurs with more aggressiveness in kidney transplant recipients compared to the general population. Immunosuppressive therapy plays a crucial role in the development of post-transplant malignancy. Concretely, cyclosporine A (CsA) has intrinsic pro-oncologic properties, while several studies report a regression of cancer after the introduction of rapamycin (RAPA). However, their effect on the extracellular vesicle (EV) content from CRC cell lines and their relevance in the pre-metastatic niche have not yet been studied. Here, we investigated the effect of RAPA and CsA in EV-miRNAs from metastatic and non-metastatic CRC cell lines and the role of relevant miRNAs transferred into a pre-metastatic niche model. EV-miRNA profiles showed a significant upregulation of miR-6127, miR-6746-5p, and miR-6787-5p under RAPA treatment compared to CsA and untreated conditions in metastatic cell lines that were not observed in non-metastatic cells. From gene expression analysis of transfected lung fibroblasts, we identified 22 shared downregulated genes mostly represented by the histone family involved in chromatin organization, DNA packaging, and cell cycle. These results suggest that EV-miR-6127, miR-6746-5p and miR-6787-5p could be a potential epigenetic mechanism induced by RAPA therapy in the regulation of the pre-metastatic niche of post-transplant colorectal cancer.

## Introduction

Cancer in solid-organ transplant recipients represents the consequence of long-term immunosuppression. The incidence and aggressiveness of each type of cancer is different in transplant recipients than in the general population, suggesting the existence of distinct mechanisms involved in oncogenesis^1–3^. Specifically, colorectal cancer (CRC) in kidney transplant recipients (KTRs) has a slightly higher incidence than that in the general population but a significantly more aggressive behaviour. Conversely, other tumours in KTRs have a higher incidence compared to the general population, but their behaviour is not as aggressive as that of CRC^4–6^.

These differences are largely due to the specific pro- and anti-neoplastic effects of the different immunosuppressive agents employed in KTRs. Two of the most commonly used drugs with different effects on carcinogenesis are rapamycin (RAPA) and cyclosporine A (CsA); while RAPA has anti-angiogenic and anti-proliferative effects, CsA promotes tumour formation and progression^7,8^. The mechanisms most affected by RAPA and CsA are the mammalian target of rapamycin (mTOR) signalling pathway^9^, the transforming growth factor beta (TGF-β) cytokine^10^, and the vascular endothelial growth factor (VEGF) protein^11^.

More specifically, RAPA, as a mTOR pathway inhibitor, has been shown to reduce cell proliferation, decrease cell cycle progression and induce apoptosis. On the other hand, while CsA has been described to activate necroptosis independently of the calcineurin pathway, its promoting effects on tumour proliferation and progression are undoubted^4,12–14^. However, even though these mechanisms explain some of the differences observed in post-transplant malignancy (PTM) incidence and behaviour, this cannot be applied to all tumours and different CRC subtypes^15,16^.

Therefore, new PTM-related mechanisms need to be explored to understand the oncogenesis and behaviour of CRC in KTRs. In this setting, extracellular vesicles (EVs) represent an intriguing mechanism of disease in oncogenesis. EVs are secreted by most cell types and are involved in intercellular communications in physiological processes as well as in tumour progression^17,18^. The importance of EVs has been largely described in the development and survival of CRC cells, including cellular transformation and proliferation^18,19^. In addition, EVs in CRC have been shown to promote tumour invasion and metastasis by changing the expression of intercellular proteins, cell matrix components and pre-metastatic niche elements^20,21^. EVs contain proteins, mRNAs, long non-coding RNAs, miRNAs, DNA fragments, lipids and other small molecules, which can modify the biological proprieties of target cells^17^. Among all the EV components, recent studies have shown the important role of miRNAs in tumour cell plasticity and angiogenesis modulation^22^, migration^23^, and immune response^24^. Several miRNAs transferred by EVs modulate CRC cells’ oncogenes^25^ or induce tumour suppression^26^.

Here, we investigated for the first time the effect of RAPA and CsA in EV-miRNAs content from metastatic and non-metastatic CRC cell lines. We have previously reported that the KRAS mutation plays a relevant role in CRC progression in renal transplant recipients^5^. From the Cancer Cell Line Encyclopedia (CCLE)^27^, we found that metastatic HCT116 and non-metastatic SW480 CRC cell lines were KRAS-mutated. Therefore, they were considered suitable to develop our study. Moreover, we studied the role of EVs in the pre-metastatic niche formation represented by a lung fibroblast cell line.

## Results

### Characterization of extracellular vesicles from HCT116 and SW480 cell lines under Rapamycin and Cyclosporine A

Our first approach was to determine a dose for RAPA and CsA to treat cells without inducing any phenotypic changes. Thus, cells were incubated with diverse concentrations of both immunosuppressants for 24 h. Cell viability, proliferation, apoptosis and the expression of different cancer stem cell markers (CD133, CD24, CD44, CD29, CD73 and CD105) were analysed. According to the dose-response curve, no differences between the diverse doses were found in both cell lines, showing that none of them was toxic (Supplementary Methods and Supplementary Figs 1S, 2S). Moreover, surface makers were not modified (Supplementary Figs 3S, 4S). Therefore, all experiments were performed with 20 nM RAPA and 10 µM CsA, the most commonly used *in vitro* concentrations for both cell lines in the literature^12,13^. Subsequently, we characterized HCT116 and SW480 by analysing EV production with and without 24 h of treatment.

**Figure 1:**
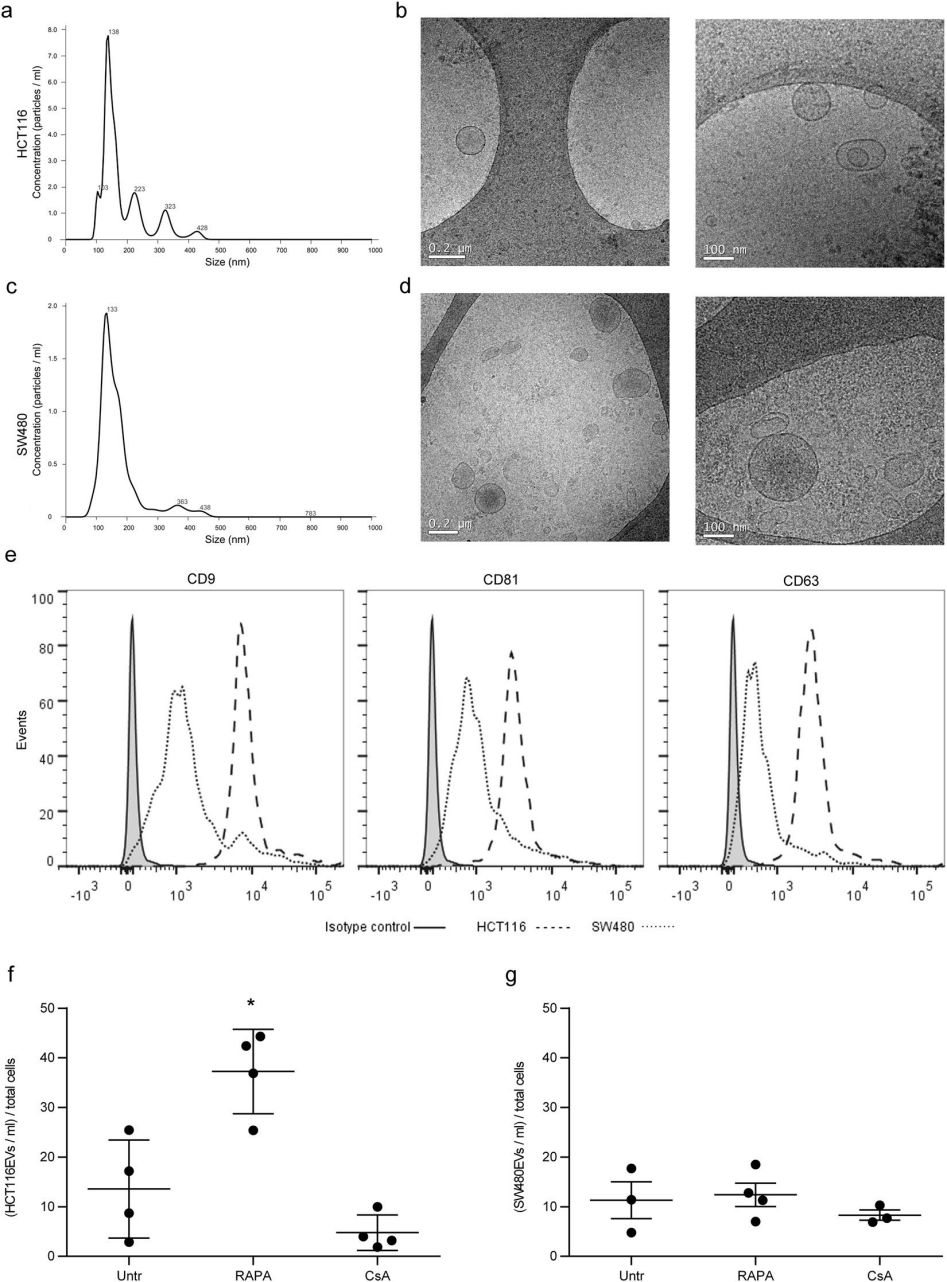
Characterization of extracellular vesicles from HCT116 and SW480 cell lines under rapamycin and cyclosporine A. Characterization of EVs released by HCT116 and SW480 CRC cell lines. (**a**,**c**) NTA measurement shows the concentration and size distribution of (**a**) HCT116EVs and (**c**) SW480EVs. (**b**,**d**) Images from cryo*-*electron microscopy of purified (**b**) HCT116EVs and (**d**) SW480EVs (scale bars 0.2 and 0.1 μm. (**e**) Bead-based flow cytometry analysis of HCT116EVs and SW480EVs stained with EV markers: CD9, CD81, and CD63. (**f**,**g**) EV production in Untr, RAPA, and CsA treatment was calculated as EVs/ml per total cells in HCT116 and SW480. Data are expressed as the mean ± SD. (n = 4). *P < 0.05 *versus* Untr (untreated) by Student’s t-test.

HCT116-derived EVs (HCT116EVs) and SW480-derived EVs (SW480EVs) were characterized by Nanoparticle Tracking Analysis (NTA; Fig. 1a,c), Transmission Electron Microscopy (TEM; Fig. 1b,d) and Bead-based Flow Cytometry (Fig. 1e). EV production was quantified using NTA and normalized to the number of producing cells. A significant increase of HCT116EVs under RAPA treatment compared to CsA and untreated cells was observed (p = 0.0004 and p = 0.010; 37.25 ± 4.25, 4.77 ± 1.79, and 13.56 ± 4.93 EVs/ml for total cells, respectively). RAPA induced a 174% increase in the number of EVs, whereas CsA induced a 64%, but not significant, decrease compared to the untreated condition. In contrast, in the SW480 cell lines, RAPA and CsA treatment did not induce any significant changes compared to the untreated cells (Fig. 1f,g).

### Differential expression of EV-miRNAs in HCT116 compared to SW480 under Rapamycin and Cyclosporine A

HCT116EVs and SW480EVs were found to contain large amounts of miRNAs by Agilent Bioanalyzer small RNA chips. EV-miRNA profiles (10 and 40 nucleotides) were observed under RAPA, CsA, and untreated conditions (Supplemental Fig. 5S).

**Figure 2:**
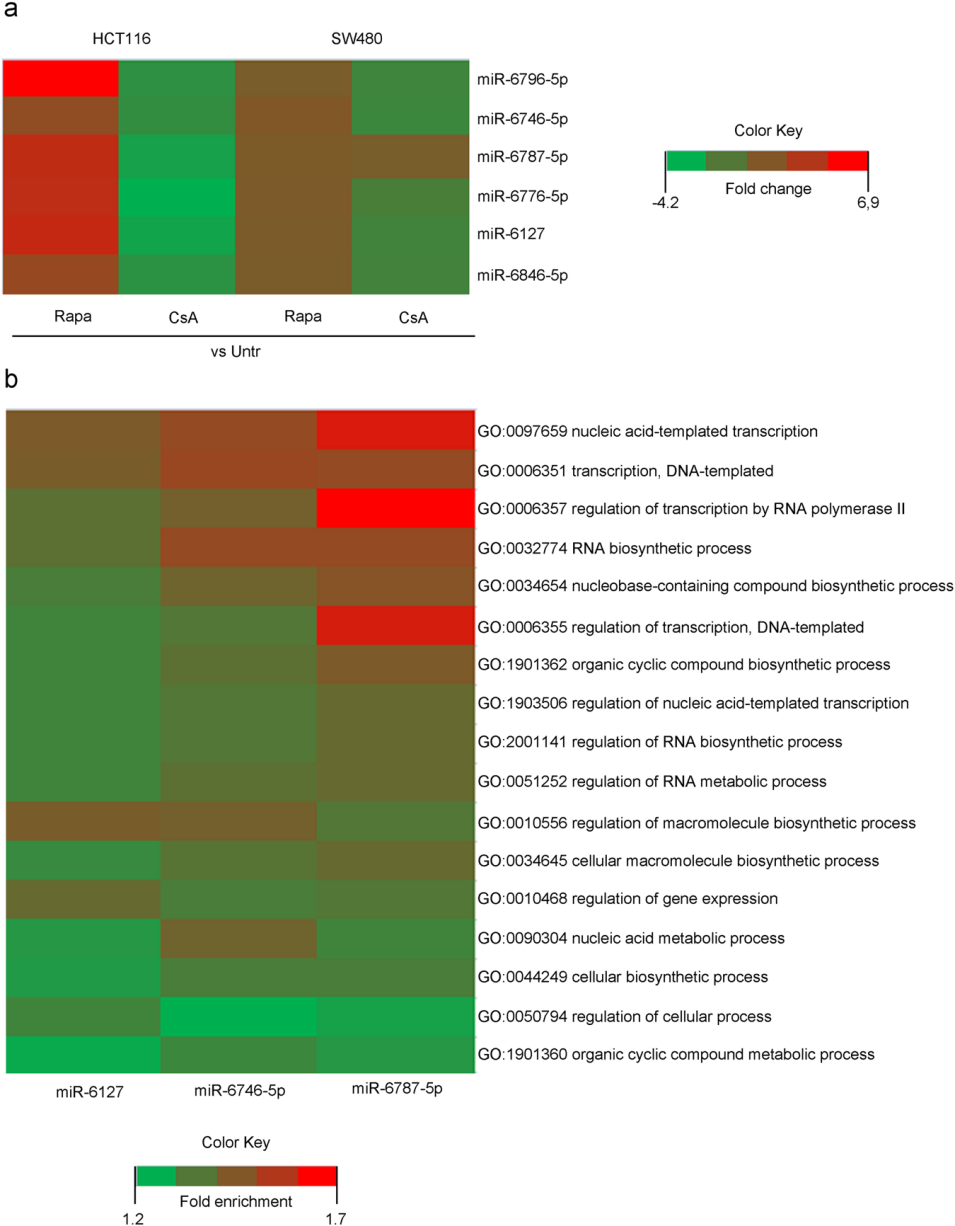
Differential expression of EV-miRNAs in HCT116 compared to SW480 under rapamycin and cyclosporine A. (**a**) Heat map of miRNA analysis. miRNA array analysis revealed six miRNAs significantly upregulated and downregulated under RAPA and CsA treatment compared to untreated cells in HCT116, respectively. No significant expression was observed in SW480 under the same conditions. Colour intensity levels indicate FC (from −4.2 to 6.9). (**b**) Heat map of Gene Ontology (GO) biological process enrichment of miR-6127, miR-6746-5p and miR-6787-5p. Gene Ontology displayed only results with false discovery rate < 0.05. Colour intensity levels indicate fold enrichment (from 1.7 to 1.2). FC = fold change.

miRNA microarray bioinformatics analysis was performed and, according to the fold change (FC), showed that 6 miRNAs were significantly enriched under RAPA treatment and impoverished under CsA in HCT116 compared to the untreated condition (Fig. 2a). A higher expression of these miRNAs under RAPA *versus* the untreated cells was also observed in SW480 cells. A lower expression under CsA *versus* untreated cells was also shown, except for miR-6787-5p. However, in the non-metastatic cell line, the expression pattern was not significant. Among the six miRNAs, miR-6127, miR-6746-5p, and miR-6787-5p were selected due to having the best validation obtained by RT-PCR (Table 1). TargetScan was used to identify target genes annotated for the miRNAs of interest, and Gene Ontology was performed to determine their function in the biological mechanisms. The main biological processes regulated by miR-6127, miR-6746-5p, and miR-6787-5p were nucleic acid-templated transcription, RNA biosynthesis and regulation of macromolecule biosynthesis. They showed a fold enrichment from 1.26 to 1.67, indicating an over-representation (>1) of the target genes involved in these biological processes (Fig. 2b).

**Table 1:** Differential expression of EV-miRNAs in HCT116 compared to SW480 under RAPA and CsA treatment.

miR-ID	HCT116				SW480				RT-PCR Validation
	RAPA vs Untr		CsA vs Untr		RAPA vs Untr		CsA vs Untr		
	FC	P	FC	P	FC	P	FC	P	
miR-6796-5p	6.978	0.001	−2.204	0.003	1.089	0.615	−1.483	0.125	Ø
miR-6746-5p	2.084	0.030	−2.024	0.005	1.423	0.034	−1.626	0.023	√
miR-6787-5p	4.166	0.035	−3.292	0.022	1.212	0.215	1.024	0.920	√
miR-6776-5p	4.126	0.017	−4.276	0.004	1.274	0.124	−1.038	0.757	Ø
miR-6127	4.457	0.007	−3.486	0.004	1.161	0.232	−1.381	0.056	√
miR-6846-5p	2.345	0.014	−2.309	0.009	1.111	0.460	−1.326	0.053	Ø

Target genes had cumulative weighted context++ scores from the top to zero.

FC = fold change.

*P < 0.01 *versus* Untr (untreated).

Ø Negative RT-PCR Validation.

√ Positive RT-PCR Validation.

### Validation of differentially expressed miRNAs in HCT116 and SW480 cell lines and their EVs with qRT-PCR

To validate the data obtained by microarray analysis, the expression of relevant miRNAs was evaluated in both cell lines and in their derived EVs by RT-PCR.

**Figure 3:**
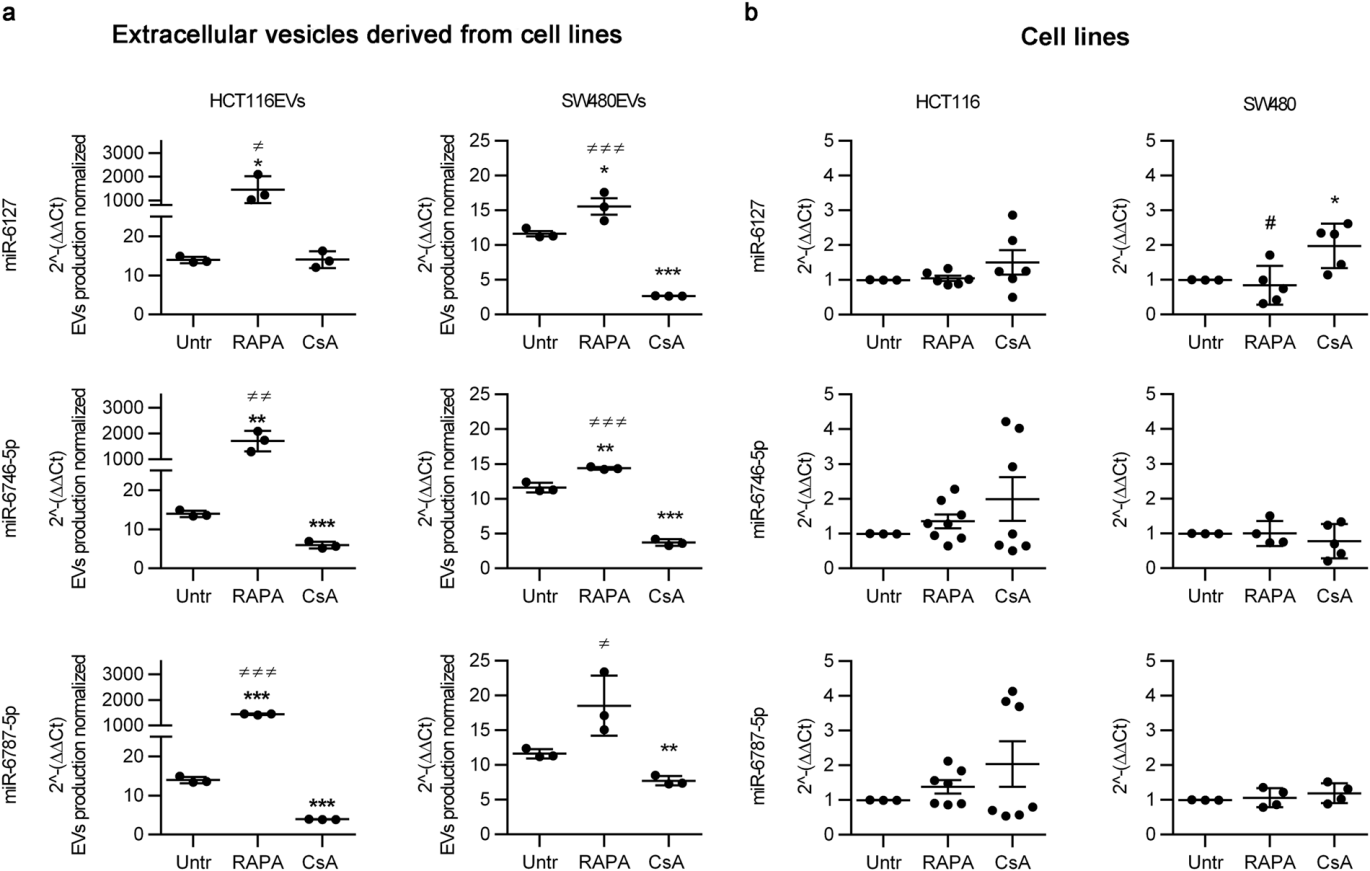
Validation of differentially expressed miRNAs in HCT116 and SW480 cell lines and their EVs with qRT-PCR. (**a**) miR-6127, miR-6746-5p, and miR-6787 relative expression in HCT116EVs and SW480EVs was normalized to EV production in Untr, RAPA, and CsA treatment. Data are expressed as the mean ± SD. (n = 3). *P < 0.05 *versus* Untr (untreated) and ≠P < 0.05 *versus* CsA treatment. (**b**) miR-6127, miR-6746-5p, and miR-6787 relative expression in HCT116 and SW480 in Untr, RAPA, and CsA treatment. Data are expressed as the mean ± SD. (n = 6). *P < 0.05 *versus* Untr (untreated) and ≠ P < 0.05 *versus* CsA by Student’s t-test.

The expression of EV-miRNAs was normalized by EV production in cell cultures. miR-6127, miR-6746-5p, and miR-6787-5p expression was significantly higher in the presence of RAPA compared to untreated conditions and CsA treatment, except for miR-6787-5p in SW480EVs. Conversely, CsA treatment induced a significant decrease in all EV-miRNA expression, except in the case of miR-6127 in HCT116EVs (Fig. 3a).

Otherwise, in both CRC cell lines, the expression of all miRNAs did not show any significant changes under drug treatment compared to untreated cells, except in the case of miR-6127 in SW480 under CsA treatment, whose expression was significantly higher compared to untreated cell cultures (p < 0.0001) (Fig. 3b).

### Epigenetic genes are transcriptionally downregulated in lung fibroblasts by miR-6127, miR-6746-5p, miR-6787-5p, and miR-mix

We investigated the effects of miR-6127, miR-6746-5p and miR-6787-5p in an *in vitro* model mimicking the pre-metastatic niche through their overexpression into a human lung fibroblast cell line (IMR90).

**Figure 4:**
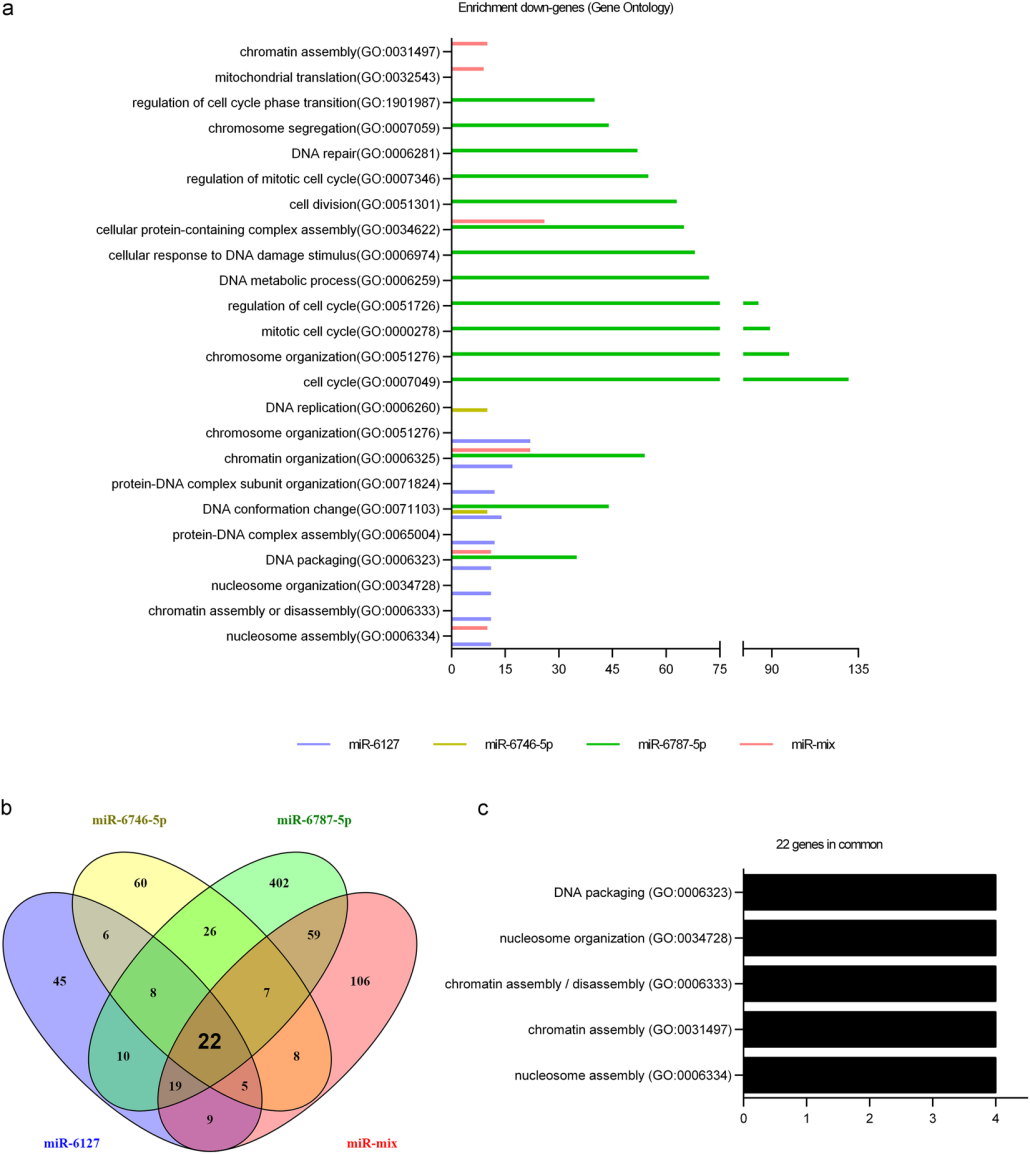
Epigenetic genes are transcriptionally downregulated in lung fibroblasts (IRM90) by miR-6127, miR-6746-5p, miR-6787-5p, and miR-mix. IRM90 was transfected with miR-6127, miR-6746-5p miR-6787-5p and miR-mix for 24 h (n = 3). (**a**) The GO-Enrichment analysis from four lists (miR-6127, miR-6746-5p and miR-6787 and miR-mix) of downregulated genes of transfected IMR90 *versus* untreated. (**b**) Venn diagram of significantly downregulated transcript lists of miRNA transfections revealed 22 genes in common. (**c**) GO terms of the common genes show biological processes mainly related to epigenetic genes. GO = Gene Ontology.

**Figure 5:**
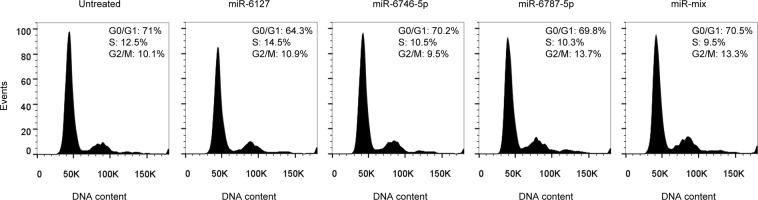
No significant changes in the cell cycle of lung fibroblasts were induced by miR-6127, miR-6746-5p, miR-6787-5p, and miR-mix. Cell cycle profiles of IRM90 untransfected and transfected with miR-6127, miR-6746-5p, miR-6787-5p, and miR-mix for 48 h (n = 3). The x-axis represents the DNA content of the nuclear population, whereas the y-axis identifies the events. The percentage of cells present in G0/G1, S, and G2/M phases are shown.

Cells were transfected with 10 pmol of miR-6127, miR-6746-5p and miR-6787-5p separately and in a mix together (miR-mix). Untransfected IMR90 cells were included as a negative control. After transfection, gene expression was evaluated by a Clariom S WT Plus array. Every transfected miRNA decreased the expression of several genes involved in different biological processes (Gene Ontology), most of them similar or closely related to each other and involved in epigenetic regulation. In particular, miR-6127 reduced the expression of genes associated with genetic packaging and organization, miR-6746-5p with DNA conformation and replication, and miR-6787-5p with DNA packaging, conformation, organization and cell cycle regulation. In transfected fibroblasts with miR-mix, chromatin organization, DNA packaging, and nucleosome assembly were the biological mechanisms affected (Fig. 4a). To visualize the shared downregulated genes between the different miRNAs and the miR-mix, a Venn diagram was generated (Fig. 4b). The results demonstrated that 12 out of 22 common genes were histone genes (HIST1H1D, HIST1H2BB, HIST1H4I, HIST1H2BG, HIST1H3I, HIST1H3J, HIST1H3H, HIST1H3B, HIST1H4A, HIST1H4F, and HIST1H3F; Table 2). Histone genes are implicated in DNA packing, nucleosome organization, chromatin assembly or disassembly, and nucleosome assembly (Fig. 4c), and all these biological mechanisms were correlated with the previous miRNA analysis (Fig. 2b). During the cell cycle, histones are crucial for efficient replication and segregation of chromosomes. Thus, we investigated the effect of miR-6127, miR-6746-5p, miR-6787-5p, and miR-mix in IMR90 during the cell cycle. However, none of the miRNAs used was able to modify the cell cycle profile of the fibroblasts (Fig. 5).

**Table 2:** Downregulated common genes from Venn diagram.

Gene ID	Official Full Name	miR-6127	miR-6746-5p	miR-6787-5p	miR-mix
		FC	FC	FC	FC
HIST4H4	histone cluster 4 H4	−1.718	−1.552	−2.181	−1.515
HIST1H3I	histone cluster 1 H3 family member i	−1.927	−1.559	−3.055	−1.852
HIST1H3J	histone cluster 1 H3 family member j	−1.929	−1.891	−3.302	−2.135
HIST1H3H	histone cluster 1 H3 family member h	−1.508	−1.547	−1.977	−1.920
HIST1H3B	histone cluster 1 H3 family member b	−1.974	−1.630	−3.060	−1.947
HIST1H4A	histone cluster 1 H4 family member a	−1.948	−2.181	−1.687	−1.687
HIST1H4F	histone cluster 1 H4 family member f	−1.622	−1.612	−3.009	−1.918
HIST1H1D	histone cluster 1 H1 family member d	−1.664	−1.532	−3.773	−1.955
HIST1H2BB	histone cluster 1 H2B family member b	−1.725	−1.567	−2.453	−2.120
HIST1H4I	histone cluster 1 H4 family member i	−1.888	−2.001	−2.882	−2.165
HIST1H2BG	histone cluster 1 H2B family member g	−1.614	−1.746	−2.623	−2.092
HIST1H3F	histone cluster 1 H3 family member f	−1.609	−1.570	−2.727	−1.783
HIST1H2AG	histone cluster 1 H2A family member g	−1.709	−1.658	−3.150	−2.095
DUSP19	dual specificity phosphatase 19	−1.742	−1.565	−1.740	−1.540
PPM1N	protein phosphatase. Mg2+/Mn2+ dependent 1N	−1.603	−1.636	−1.582	−1.546
RPL22L1	ribosomal protein L22 like 1	−1.640	−1.505	−3.187	−1.741
GBP1	guanylate binding protein 1	−1.589	−1.689	−2.198	−1.511
TIMM10	translocase of inner mitochondrial membrane 10	−1.668	−1.567	−1.871	−1.722
STEAP1	STEAP family member 1	−1.539	−2.226	−2.436	−1.794
NXT2	nuclear transport factor 2 like export factor 2	−1.588	−1.784	−2.269	−1.670
ZDHHC11B	zinc finger DHHC-type containing 11B	−1.612	−2.992	−2.401	−3.074
GPR65	G protein-coupled receptor 65	−1.630	−1.960	−2.290	−1.559

miR-mix = mix of miR-6127, miR-6746-5p, and miR-6787-5p.

FC = fold change.

## Discussion

In kidney transplant recipients (KTR), colorectal cancer (CRC) is more aggressive than in the general population, with a high tendency to metastasize^6,8^. It is characterized by primary and metastasis-tumour specific miRNA expression^28,29^. and an association between the upregulation^30,31^ and the downregulation^32,33^ of many miRNAs with oncogenesis and tumour progression has been described^34^. For instance, miRNAs can inhibit cancer development by targeting mTORC1 or affecting other genes associated with the mTOR signalling pathway involved in proliferation, invasion, apoptosis and the cell cycle^35–37^. Cancer-derived miRNAs exert all these effects on tumour microenvironment and distant organs by means of extracellular vesicles (EVs). Cancer-derived EVs are indeed an efficient way for neoplastic cells to communicate with each other within the primary tumour or with the pre-metastatic niche^22–24,38–40^. Apart from their role as regulatory biomolecules, miRNA signature is also specific for each cancer type^41,42^. An important point is that according to cell type and drug treatments, the molecular composition of EVs can significantly change^43,44^.

Two of the most commonly used drugs in kidney transplantation, RAPA and CsA, have a distinct role in cancer behaviour due to their antagonistic oncogenic properties. Both of them induce phenotypic and gene expression changes in metastatic cancer cell lines, as well as affect cell cycle, proliferation, and invasiveness^10,45^. However, the role of cancer-derived EVs under immunosuppressive therapy has not yet been described. In this study, for the first time, we evaluated the impact of RAPA and CsA on the content of CRC cell line-derived EVs and their influence on the pre-metastatic niche.

We showed that EV production was different depending on the metastatic nature of the CRC cells and the drug used. RAPA treatment induced an increase in EV release from the metastatic CRC cell line compared to the untreated condition, whereas CsA treatment showed a tendency to decrease EV production. This difference may be explained by taking into account the well-known effects of the mTOR signalling pathway in protein synthesis and cell growth that integrate both intracellular and extracellular mitogenic signals. Thus, it is likely that mTOR inhibition by RAPA could give rise to a signal transduction cascade for cell cycle arrest reflected in an increase in EV production.

Furthermore, we observed a differential miRNA expression profile of EV-miRNAs under immunosuppressive treatment depending on the metastatic nature of the CRC cell line and the drug used. RAPA treatment induced a significant upregulation of miR-6127, miR-6746-5p and miR-6787-5p in the metastatic CRC cell line compared to the untreated condition, whereas CsA treatment showed a downregulated expression of these three miRNAs. The RT-PCR data confirmed these results and showed that the miRNA expression under RAPA *versus* untreated cells was at least 100 times higher in HCT116 than in SW480, suggesting the involvement of these miRNAs in the metastatic process. Nucleic acid-templated transcription, RNA biosynthesis, and regulation of macromolecule biosynthesis were the biological processes predicted from these miRNAs in the bioinformatic analysis. Nevertheless, the expression of these EV-miRNAs did not resemble those of their parent cells, supporting the existence of a selective and specific loading mechanism for miRNAs into EVs^46,47^.

When lung fibroblasts were stimulated with these miRNAs, they showed a significant downregulation of several epigenetic genes involved in chromatin organization, DNA packaging and nucleosome assembly compared to the untransfected condition. Our results suggest that RAPA may induce the overexpression of these EV-miRNAs as a potential epigenetic regulation of the pre-metastatic niche in one of the preferred host organs for CRC. Among the surrounding tumour cells, fibroblasts represent a predominant cell type, and it has been described that EVs produced by cancer cells are able to differentiate fibroblasts into myofibroblasts^48^. After the transfection of all miRNAs into fibroblasts, we observed a reduction of transcripts involved in epigenetic regulation compared to untreated controls, including chromatin assembly and organization, DNA replication, and cell cycle regulation. A total of 22 downregulated genes were identified from the analysis of transfected fibroblasts. Interestingly, histone genes (HIST1H1D, HIST1H2BB, HIST1H4I, HIST1H2BG, HIST1H3I, HIST1H3J, HIST1H3H, HIST1H3B, HIST1H4A, HIST1H4F, and HIST1H3F) were the most representative type. Histones have been shown to play a crucial role in DNA packaging, cell replication, and segregation and recombination of chromosomes. mTORC1 signalling regulates the occurrence of histone post-translational modification and therefore the control of DNA transcription and correct RNA processing^49^. Moreover, several studies have shown that RAPA modulates chromatin structure and function, inducing lower protein synthesis and growth^50^. We propose that EV-miRNAs overexpressed under RAPA treatment may reflect the well-established anti-neoplastic properties of this drug. Thus, we hypothesize that the EV enrichment of miR-6127, miR-6746-5p and miR-6787-5p could give rise to epigenetic mechanisms in the pre-metastatic niche likely associated with the drug tumour suppression function.

Moreover, it has also been described that epigenetic changes influence the transition of fibroblasts into myofibroblasts that are relevant in cancer progression^51^. Therefore, we hypothesize that these EV-miRNAs may be implicated in the inhibition of fibroblast activation. Further studies are needed to determine the molecular mechanisms by which miR-6127, miR-6746-5p and miR-6787-5p downregulate the histone genes modulating the pre-metastatic niche.

## Methods

### Cell culture

SW480 and IRM90 were cultured in DMEM-F12 and HCT116 in McCoy’s 5 A (Gibco), supplemented with 10% (v/v) foetal bovine serum (FBS; Gibco) and 1% (v/v) penicillin/streptomycin (Biological Industries). SW480 and HCT116 were treated 24 h with 20 nM of RAPA (Sigma-Aldrich) and 10 µM CsA (LC Laboratories). All cells were maintained at 37 °C in a humidified incubator with 5% CO_2_. Cell lines were kindly provided by the IMIM (Institut Hospital del Mar d’Investigacions Mèdiques).

### Extracellular vesicle isolation

EVs were isolated from cells cultured in EV-depleted FBS (Solmeglas) using sequential centrifugations at 800 g for 7 min and 2,000 g for 12 min. Supernatants were filtered through a 0.1 μm pore filter and ultracentrifuged (Optima L100XP, Beckman) at 100,000 g for 2 h. A PBS washing step was performed followed by a second ultracentrifugation. Pellets were resuspended in PBS (n = 3 per group).

The manuscript follows the ISEV guidelines for extracellular vesicle characterization^52^ (Supplemental Material).

### Transmission electron microscopy (TEM)

A Holey Carbon support film on a 400-mesh copper grid was used. After glow discharge, the sample was deposited onto the grid, which was mounted on a plunger (Leica EM-CPC) and blotted with Whatman No. 1 filter paper. The suspension was vitrified by rapid immersion in liquid ethane. The grid was mounted on a Gatan 626 cryo-transfer system and inserted into the microscope. Images were obtained using a Jeol JEM 2011 cryo-electron microscope operated at 200 kV, recorded on a Gatan Ultrascan US1000 CCD camera and analysed with a Digital Micrograph 1.8 (n = 3 per group).

### Nanoparticle tracking analysis (NTA)

The size distribution and concentration of EVs were measured using a NanoSight LM10 instrument (Malvern), equipped with a 638 nm laser and CCD camera (model F-033). Data were analysed with NTA Software version 3.1 (Build 3.1.46). Samples were evaluated in PBS (n = 3 per group).

### Flow cytometry

EVs were coupled to 4 µm aldehyde/sulfate-latex microspheres (Invitrogen) for 15 min at RT, resuspended in BCB buffer (PBS/0.1% BSA/0.01% NaN_3_; Sigma-Aldrich) and incubated overnight. Samples were centrifuged at 2,000 g for 10 min, washed with BCB buffer and re-suspended in PBS after a second RT centrifugation at 2,000 g for 10 min. EV-coated beads were then labelled at 4 °C with anti-CD9-FITC-conjugated (MA1-19557, ThermoFisher), CD63-Alexa Fluor 488 (MEM-259, ThermoFisher), anti-CD81-PE-conjugated (A15781, ThermoFisher) and polyclonal IgG isotype (IgG1-PE MA1-10415, IgG1-FITC MA1-10413, ThermoFisher) antibodies for 30 min. After BCB washing, EV-coated beads were acquired with a BD LSR Fortessa (BD Biosciences). A total of 10,000 beads/events were acquired for each sample (n = 3 per group).

IMR90 was fixed with 70% ethanol overnight at −20 °C. After washing with PBS, propidium iodide and RNAse were used for 30 min of DNA staining. Cells were acquired with CANTO II (BD Biosciences).

All data were analysed with FlowJo software (Tree Star) (n = 3 per group).

### Isolation and quantification of EV-RNAs

RNA was extracted using a miRNeasy Mini KIT (Qiagen) according to the manufacturer’s instructions. Yield and size distribution were analysed using an Agilent 2100 Bioanalyzer with an RNA 6000 Pico kit (Agilent Technologies). Concentration was measured by a Qubit*®* RNA HS Assay Kit (Life Technologies, Thermo Fisher Scientific Inc.).

### miRNA Array

RNA samples were labelled using a FlashTag™ Biotin HSR RNA Labeling Kit (Affymetrix). The process begins with a brief tailing reaction followed by ligation of the biotinylated signal molecule to the target RNA sample. Afterward, the biotin-labelled RNA was hybridized onto a GeneChip miRNA 4.0 Array for 42 h at 49 °C using an Affymetrix Hybridization Oven. Using the Affymetrix GeneChip system, GeneTitan Arrays were washed and stained in an Affymetrix Fluidics Station 450 and scanned using an Affymetrix GeneChip Scanner 3000 System. The data were analysed with Expression Console Software using RMA analysis. A filtering step excluding probes not reaching the lower quartile of the coefficient of variation was employed, and the total number of obtained human miRNAs was 4993.

### Bioinformatics analysis

Relevant miRNAs were selected based on the fold change (FC = 1.5) and the p-value (p < 0.01). Target Scan (www.targetscan.org) was used to identify target genes with a cumulative weighted context++score from the top to zero. Gene Ontology (www.geneontology.org) was used to find the main biological processes with a Fold Enrichment from 1.26 to 1.67. For the Affymetrix miRNA 4.0 GENE chip array, we selected the genes significantly downregulated with the transfection *versus* control.

### Analysis of miRNA expression by RT-PCR

Total RNA was extracted using TRIzol reagent (Invitrogen) following the manufacturer’s instructions. cDNA was generated from 200 ng of total RNA from cell culture and from EVs using the miScript II RT Kit (Qiagen). All primers are listed in Supplementary Table S1. RNU6B was used to normalize (n = 3 per group).

### miRNA transfection

IMR90 was transfected with miR-6127, miR-6746-5p and miR-6787-5p separately and miR-mix, according to the manufacturer’s protocol. Briefly, cells were incubated with a mix of Opti-MEM medium (Life Technologies, Thermo Fisher Scientific Inc.) containing 10 pmol of miRNA and Lipofectamine 2,000 (Life Technologies, Thermo Fisher Scientific Inc.). After 24 h total RNA was extracted with TRIzol reagent (Invitrogen) (n = 3 per group).

### Gene Expression Array

Processing of RNA samples, fragmentation, and labelling of ss-cDNA was prepared according to the Affymetrix WT PLUS Reagent Kit user guide. Then, ss-cDNAs were hybridized for 17 h at 45 °C on a GeneChip Human Clariom S array plate using an automated GeneTitan System, which includes a hybridization oven, Fluidic Station and Scanner. Data were analysed with Expression Console Software using RMA analysis. Affymetrix gene expression data were normalized with the robust multiarray algorithm^53^ using a custom probe set definition that maps probes directly to Entrez Gene Ids (ClariomSHuman_Hs_ENTREZG)^54^. A filtering step excluding probes not reaching the lower quartile of the coefficient of variation was employed, and the total number of obtained probes was 13959.

### Differential expression

A linear model was fitted to the data, and empirical Bayes moderated statistics were calculated using the limma package (Bioconductor). Adjustment of p-values was performed by the determination of false discovery rates (FDR) using the Benjamini-Hochberg procedure^55^. All computations were performed using R statistical software. Genes representing a FC of 1.5 or greater and a moderated p-value < 0.05 were considered differentially expressed.

### Information and array data

Microarray raw data (cel files) and processed data have been deposited in the National Center for Biotechnology Information (NCBI)’s Gene Expression Omnibus and are accessible through GEO Series accession number GSE123710.

### Statistical analysis

All *in vitro* data were analysed using Student’s t-test with GraphPadPrism 6 statistical software (GraphPad Software Inc). *P < 0.05, **P < 0.01. Data are expressed as the mean ± SD.

## Supplementary information

Supplementary information
