# Supplementary material for: Effect of immunosuppression in miRNAs from extracellular vesicles of colorectal cancer and their influence on the pre-metastatic niche
Source: Sci Rep. 2019 Aug 1;9:11177. doi: 10.1038/s41598-019-47581-y (PMC6672014; doi:10.1038/s41598-019-47581-y)
Supplement: Supplementary file 1 — Supplementary information [file 41598_2019_47581_MOESM1_ESM.pdf]

Effect of immunosuppression in miRNAs from extracellular vesicles of colorectal cancer and their influence on the pre-metastatic niche

Scientific Reports

Valeria Tubita, Joan Segui-Barber, Juan José Lozano, Elisenda Banon-Maneus, Jordi Rovira, David Cucchiari, Daniel Moya-Rull, Federico Oppenheimer, Hernando Del Portillo, Josep M Campistol, Fritz Diekmann, Maria José Ramirez-Bajo and Ignacio Revuelta.

Corresponding authors: Ignacio Revuelta, MD, PhD and María José Ramírez-Bajo, PhD, Department of Nephrology and Renal Transplantation, Hospital Clinic de Barcelona, Villarroel 170 (Escala 10 – Planta 5), 08036 Barcelona, Spain, Tel.: +34-932274410/3474, Email: irevuelt@clinic.cat, mramire1@clinic.cat.

SUPPLEMENTARY MATERIAL

- Supplemental methods
- Supplemental table
- Supplemental figures
- MISEV2018 checklist

• **Supplemental methods**

Viability and proliferation assays

SW480 and HCT116 cells were seeded in a 96-well plate and treated with CsA (2, 5 and 10 µM) and RAPA (10, 20, 50 nM) or untreated for 24h. Viability and proliferation assays were performed with MTT assay (Sigma-Aldrich) and CyQuant assay kit (Molecular Probes, Invitrogen), respectively according to the manufacturer's instructions. (n=3 per group).

Annexin V/PI staining

Late and early apoptosis were evaluated through Annexin V and propidium iodide (PI). Briefly, HCT116 and SW480 were treated with CsA (2, 5 and 10 µM) and RAPA (10, 20, 50 nM) or untreated for 24h. Cells were pelleted by centrifugation (300g, 5 min) and re-suspended in annexin V binding buffer (10 mM HEPES, 140 mM NaCl, 2.5 mM $CaCl_2$), containing 1 µg/ml CY5.5-annexin V (BD Biosciences,) and 10µg/ml propidium iodide (Sigma-Aldrich) for 15 min at room temperature in the dark. Subsequently, cells were analyzed by flow cytometry, using CANTO II (BD Biosciences). A total of 10,000 beads/events were acquired for each sample. All data were analyzed with FlowJo software (Tree Star).

Flow cytometry analysis for cancer stem cells markers

For surface marker staining, cell preparations were incubated with antibodies anti-CD133-PE-conjugated (AC133, Milteny Biotec), CD24-APC (SN3 A5-2H10, eBioscience), CD29-APC-conjugated (MAR4, BD Pharmingen), CD44-PE-conjugated (515, BD Pharmingen), CD73-PE-conjugated (AD2, Milteny Biotec), and CD105APC-conjugated (43A4E1, Milteny Biotec) at 4ºC for 15 min. The labeled cells were washed in MACS buffer (2 mM EDTA, 1% (w/v) BSA in PBS), and then acquired with with CANTO II (BD Biosciences). A total of 10,000 beads/events were acquired for each sample. (n=2-6 per group). All data were analyzed with FlowJo software (Tree Star) to calculate the percentage of positive cells and Mean fluorescence intensity (FMI).

- **Supplemental table**

Table S1 Primers for RT-PCR

miR-ID	Primer Sequence
miR-6127	UGAGGGAGUGGGUGGGAGG
miR-6746-5p	UGGCGGGGGUAGAGCUGGCUGC
miR-6787-5p	UGGCGGGGGUAGAGCUGGCUGC
RNU6B	CGC AAG GAT GAC ACG CAA

• **Supplemental figures**

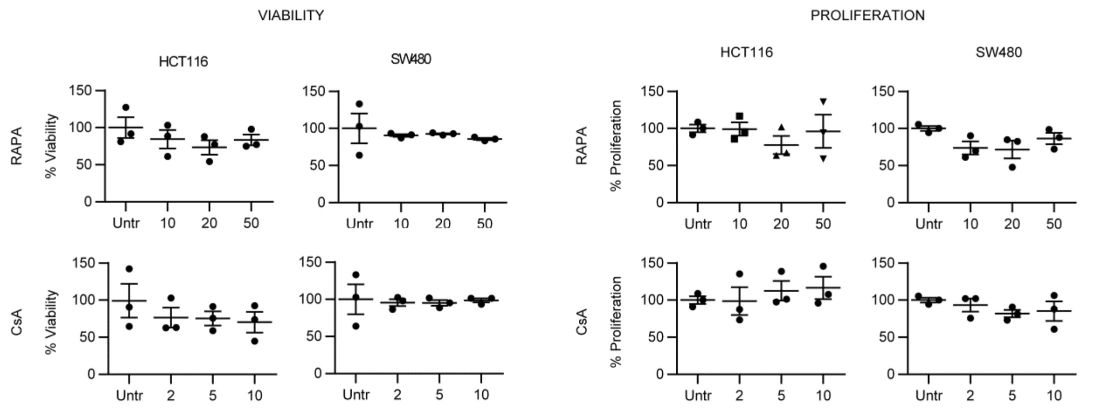

Suppl Fig. S1 Cell viability and proliferation assay for HCT116 and SW480 incubated with 2, 5 and 10 µM of CsA and 10, 20 and 50 nM of RAPA for 24 h (n=3 per group).

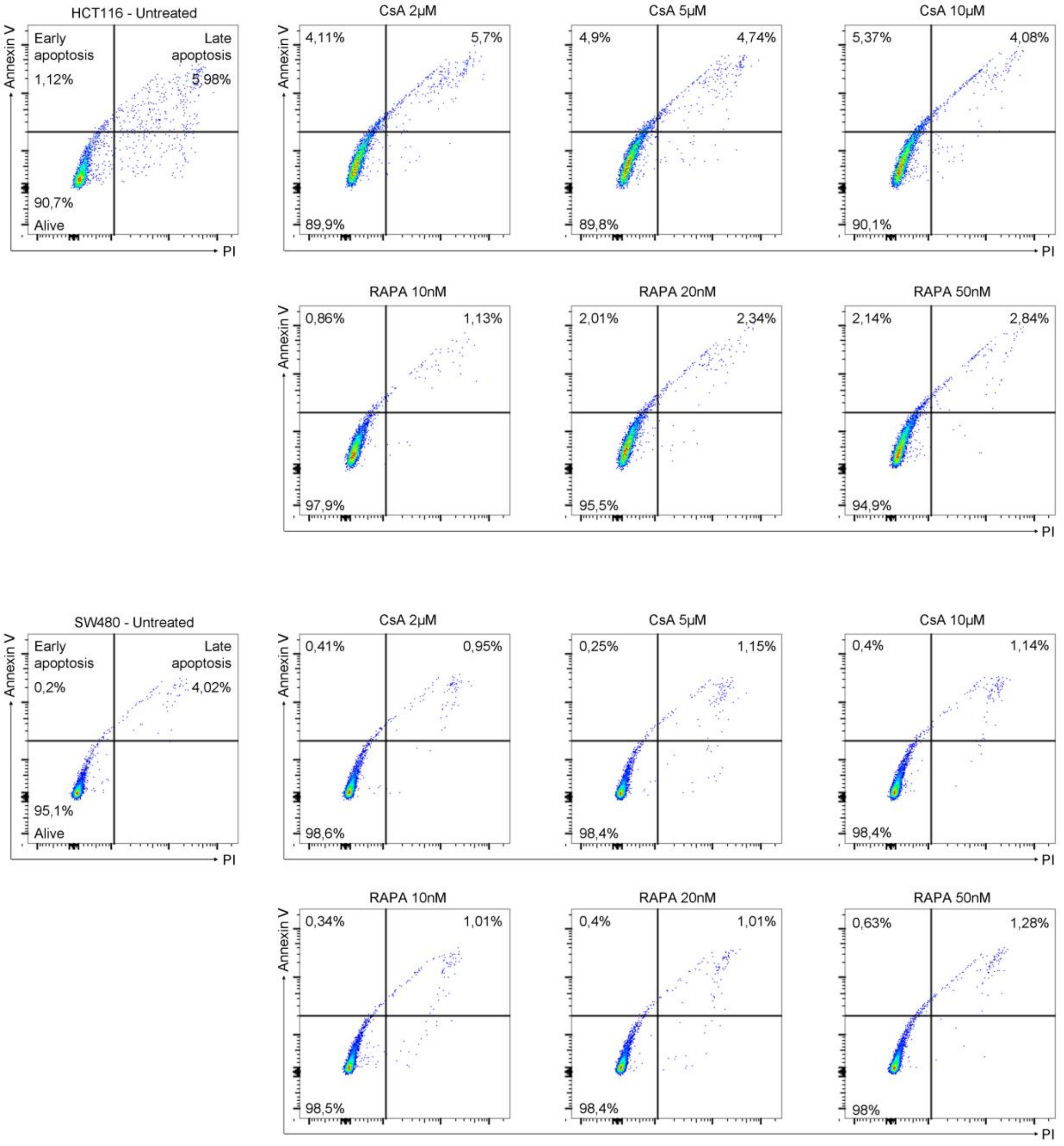

Suppl Fig. S2 Early apoptotic cells (Annexin V$^+$ / PI$^-$) and late apoptotic cells (Annexin V$^+$ / PI$^+$) were analyzed by annexin V/propidium iodide (PI) staining in HCT116 and SW480. Both cell lines were incubated with and without 2, 5 and 10 µM of CsA and 10, 20 and 50 nM of RAPA for 24 h (n=1 per group).

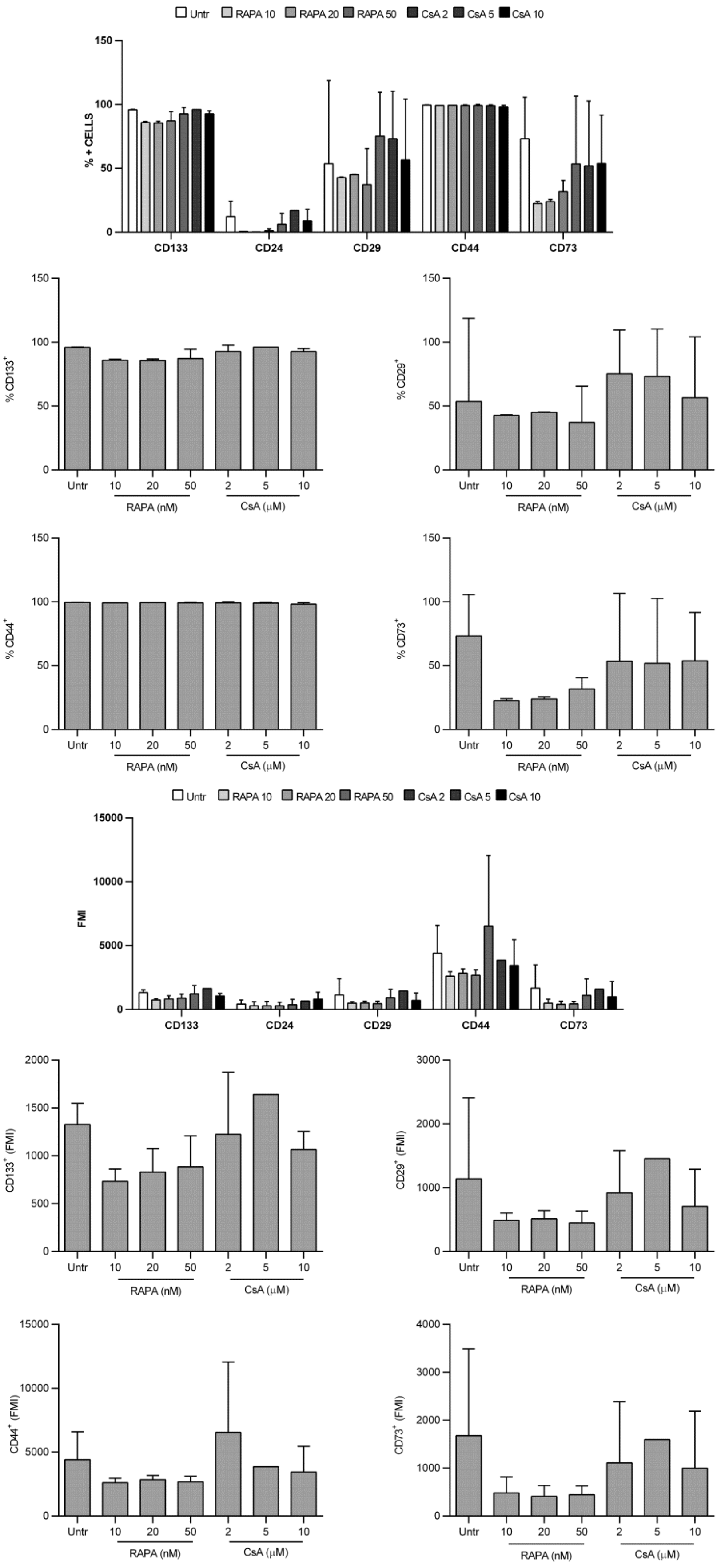

Untr
RAPA 10
RAPA 20
RAPA 50
CsA 2
CsA 5
CsA 10
% + CELLS
CD133
CD24
CD29
CD44
CD73
% CD133+
% CD29+
% CD44+
% CD73+
RAPA (nM)
CsA (μM)
FMI
CD133+ (FMI)
CD29+ (FMI)
CD44+ (FMI)
CD73+ (FMI)

Suppl Fig. S3 Cancer stem cell markers (CD133, CD24, CD29, CD44 and CD73) expression were analyzed by flow cytometry in HTC116 incubated with and without 2, 5 and 10 μM of CsA and 10, 20 and 50 nM of RAPA for 24 h (n=2-6 per group). The data obtained are represented as percentage of positive cells and Mean fluorescence intensity (FMI).

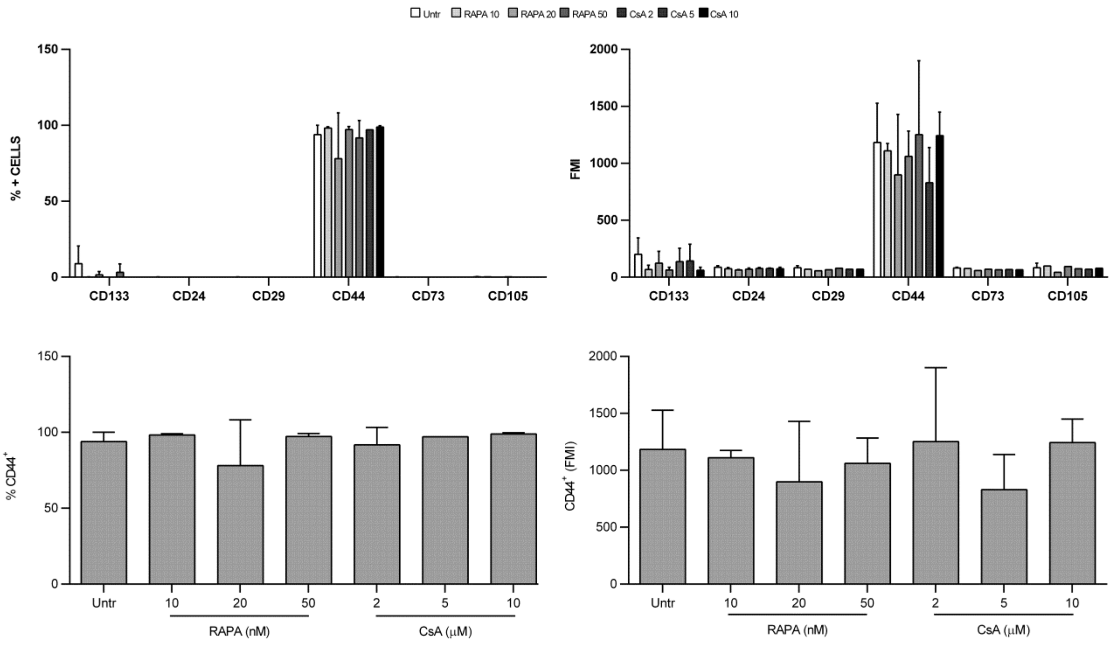

Suppl Fig. S4 Cancer stem cell markers (CD133, CD24, CD29, CD44, CD73 and CD105) expression were analyzed by flow cytometry in SW480 incubated with and without 2, 5 and 10 µM of CsA and 10, 20 and 50 nM of RAPA for 24 h (n=2-6 per group).

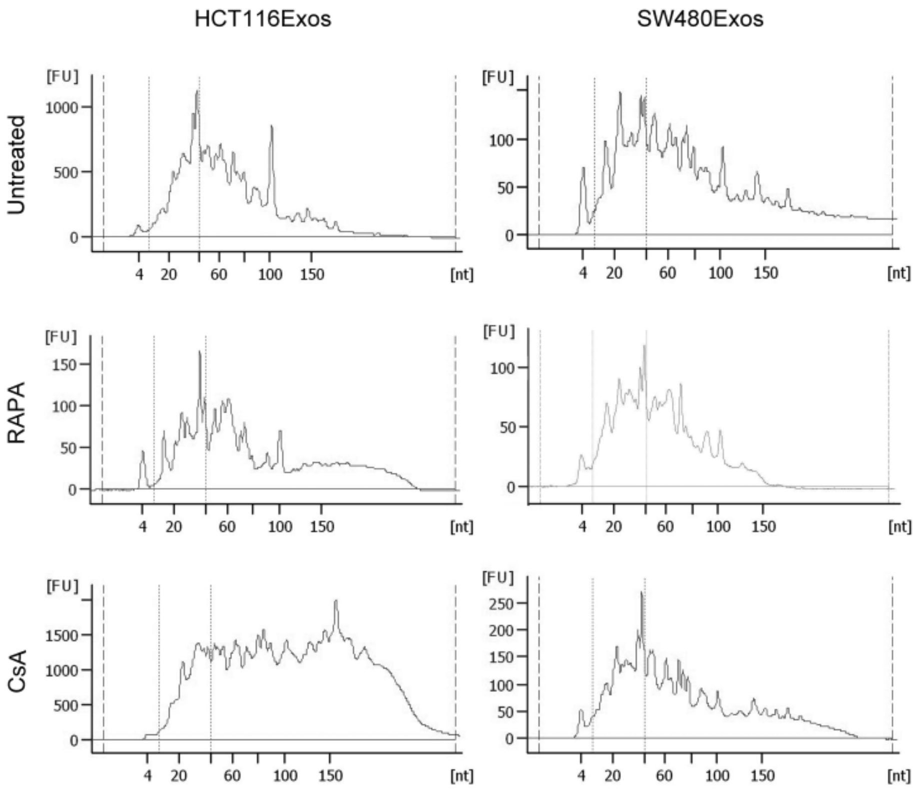

Suppl Fig. S5 Bioanalyzer analysis of EVs total RNA from HCT116Exos and SW480Exos without and with RAPA, and CsA treatment. EVs-RNA isolated was analyzed using Agilent 2100 Bioanalyzer. (n=3 per group). The electropherograms show the size distribution in nucleotides (nt) and fluorescence intensity (FU) of total RNA.

• **MISEV2018 checklist**

■ Done

1-Nomenclature

Mandatory

- Generic term extracellular vesicle (EV): With demonstration of extracellular (no intact cells) and vesicular nature per these characterization (Section 4) and function (Section 5) guidelines OR
- Generic term, e.g., extracellular particle (EP): no intact cells but MISEV guidelines not satisfied

Encouraged (choose one)

- Generic term extracellular vesicle (EV) + specification (size, density, other)
- Specific term for subcellular origin: e.g., ectosome, microparticle, microvesicle (from plasma membrane), exosome (from endosomes), with demonstration of the subcellular origin
- Other specific term: with definition of specific criteria

2-Collection and pre-processing

Tissue Culture Conditioned medium (CCM, Section 2-a)

General cell characterization (identity, passage, mycoplasma check…). Medium used before and during collection (additives, serum, other)

- exact protocol for depletion of EVs/EPs from additives in collection medium
- Nature and size of culture vessels, and volume of medium during conditioning
  A T175 flask with 15ml of medium was used during conditioning
- specific culture conditions (treatment, % O2, coating, polarization…) before and during collection
- Number of cells/ml or /surface area and % of live/dead cells at time of collection (or at time of seeding with estimation at time of collection)
  $5x10^6$ cells/15ml were seeded in a T175 per condition with estimation at time of collection of ± $25x10^6$ cells and ±97% of live cells.
- Frequency and interval of CM harvest
  24 h.

Storage and recovery (Section 2-d)

- Storage and recovery (e.g., thawing) of CCM, biofluid, or tissue before EV isolation (storage temperature, vessel, time; method of thawing or other sample preparation)
  The CCM was stored at 4˚C before starting the experiments. After 24 h, the recovered CCM was used at 4°C during sequential centrifugations.
- Storage and recovery of EVs after isolation (temperature, vessel, time, additive(s)…)
  After EVs isolation, samples were used immediately or stored during only one night at 4°C for the following applications.

3-EV separation and concentration

Experimental details of the method

- Centrifugation: reference number of tube(s), rotor(s), adjusted k factor(s) of each centrifugation step (= time+ speed+ rotor, volume/density of centrifugation conditions), temperature, brake settings

  Reference number of tubes: Polypropylene Centrifuge Tubes, Beckman Coulter 337986.
  Each tube contained 30ml of CCM.
  Rotor: SW32Ti
  Centrifugation steps:
  - 800 g for 7 min at 4˚C
  - 2,000 g for 12 min at 4˚C
  - Supernatants filtered through 0.1 µm pore filter
  - Samples ultracentrifuged (Optima L100XP, Beckman) at 100,000 g for 2 h at 4˚C
  - PBS washing step
  - Samples ultracentrifuged (Optima L100XP, Beckman) at 100,000 g for 2 h at 4˚C

4-EV characterization

Quantification (Table 2a, Section 4-a)

- Volume of fluid, and/or cell number, and/or tissue mass used to isolate EVs NTA
  30 ml of CCM were used to isolate EVs for NTA
- Global quantification by at least 2 methods: protein amount, particle number, lipid amount, expressed per volume of initial fluid or number of producing cells/mass of tissue
- Ratio of the 2 quantification figures
  It has already been shown in the Figure 1 (f,g).

Global characterization (Section 4-b, Table 3)Citometria y los marcadores

- Transmembrane or GPI-anchored protein localized in cells at plasma membrane or endosomes
  The CD63 marker was observed by Flow Cytometry
- Cytosolic protein with membrane-binding or -association capacity
  The CD9 and CD81 markers were observed by Flow Cytometry
- Assessment of presence/absence of expected contaminants
  A total absence of contaminants was observed by Electron Microscopy

(At least one each of the three categories above)

- Presence of proteins associated with compartments other than plasma membrane or endosomes
  No presence of proteins was observed.
- Presence of soluble secreted proteins and their likely transmembrane ligands
- Topology of the relevant functional components (Section 4-d)

Single EV characterization (Section 4-c)

- Images of single EVs **by wide-field and close-up**: e.g. electron microscopy, scanning probe microscopy, super-resolution fluorescence microscopy

- Non-image-based method analyzing large numbers of single EVs: NTA, TRPS, FCS, high-resolution flow cytometry, multi-angle light-scattering, Raman spectroscopy, etc.

Reporting

- Submission of methodologic details to EV-TRACK (evtrack.org) with EV-TRACK number provided (strongly encouraged)
- Submission of data (proteomic, sequencing, other) to relevant public, curated databases or open-access repositories
  Microarray raw data [.cel files] and processed data have been deposited in the National Center for Biotechnology Information [NCBI] ’s Gene Expression Omnibus and are accessible through GEO Series accession number GSE123710.
- Data submission to EV-specific databases (e.g., EVpedia, Vesiclepedia, exRNA atlas)
- Temper EV-specific claims when MISEV requirements cannot be entirely satisfied (Section 6-b)
